# Dynamic assessment of left ventricular coupling and myocardial reserve in patients with cardiogenic shock

**DOI:** 10.1093/ehjopen/oeae072

**Published:** 2024-08-26

**Authors:** Anthony J Kanelidis, Michael J Randazzo, Sara Kalantari, Bryan Smith, Ann Nguyen, Ben B Chung, Stanley Swat, Nitasha Sarswat, Christopher Salerno, Valluvan Jeevanandam, Gene Kim, Mark N Belkin, Jonathan Grinstein

**Affiliations:** Department of Medicine, Section of Cardiology, University of Chicago Medical Center, 5841 S. Maryland Ave, Room A621, Chicago, IL 60637, USA; Department of Medicine, Section of Cardiology, University of Chicago Medical Center, 5841 S. Maryland Ave, Room A621, Chicago, IL 60637, USA; Department of Medicine, Section of Cardiology, University of Chicago Medical Center, 5841 S. Maryland Ave, Room A621, Chicago, IL 60637, USA; Department of Medicine, Section of Cardiology, University of Chicago Medical Center, 5841 S. Maryland Ave, Room A621, Chicago, IL 60637, USA; Department of Medicine, Section of Cardiology, University of Chicago Medical Center, 5841 S. Maryland Ave, Room A621, Chicago, IL 60637, USA; Department of Medicine, Section of Cardiology, University of Chicago Medical Center, 5841 S. Maryland Ave, Room A621, Chicago, IL 60637, USA; Department of Medicine, Section of Cardiology, University of Chicago Medical Center, 5841 S. Maryland Ave, Room A621, Chicago, IL 60637, USA; Department of Medicine, Section of Cardiology, University of Chicago Medical Center, 5841 S. Maryland Ave, Room A621, Chicago, IL 60637, USA; Department of Surgery, Section of Cardiac Surgery, University of Chicago Medical Center, 5841 S. Maryland Ave, Room A621, Chicago, IL 60637, USA; Department of Surgery, Section of Cardiac Surgery, University of Chicago Medical Center, 5841 S. Maryland Ave, Room A621, Chicago, IL 60637, USA; Department of Medicine, Section of Cardiology, University of Chicago Medical Center, 5841 S. Maryland Ave, Room A621, Chicago, IL 60637, USA; Department of Medicine, Section of Cardiology, University of Chicago Medical Center, 5841 S. Maryland Ave, Room A621, Chicago, IL 60637, USA; Department of Medicine, Section of Cardiology, University of Chicago Medical Center, 5841 S. Maryland Ave, Room A621, Chicago, IL 60637, USA

**Keywords:** Left ventricular coupling, Myocardial reserve, Cardiogenic shock, Milrinone

## Abstract

**Aims:**

Pulmonary artery catheter haemodynamics are associated with improved survival in cardiogenic shock (CS). We investigated the utility of aortic pulsatility index (API) and cardiac power output (CPO) as surrogates for left ventricular (LV) coupling and myocardial reserve, respectively, in patients with CS undergoing dynamic assessment after a milrinone bolus.

**Methods and results:**

Patients with SCAI Stage C CS underwent a milrinone drug study (50 mcg/kg bolus infused over 10 min) to assess inotropic response. Haemodynamic measurements were obtained at baseline and following the bolus. Aortic pulsatility index and CPO were used to risk-stratify patients with the incidence of LV assist device (LVAD), orthotopic heart transplantation (OHT), or death at 1 year as the primary composite endpoint. Two hundred and twenty-four patients in SCAI Stage C CS underwent haemodynamics prior to milrinone bolus, and 117 patients had low baseline API < 1.45. Of the 117 patients, 88 had a final API < 2.2 after milrinone load, consistent with LV decoupling, in which 73% met the composite endpoint. The remaining 29 patients had a final API ≥ 2.2 consistent with LV recoupling, and only 55% met the composite endpoint (*P* = 0.046). Of the 117 patients, 40 patients had low myocardial reserve (final CPO < 0.77 W), in which 78% met the composite endpoint. Of the 77 patients who demonstrated myocardial reserve (final CPO ≥ 0.77 W), only 64% met the composite endpoint (*P* = 0.039).

**Conclusion:**

The use of API and CPO in a dynamic assessment after provocative testing led to improved risk stratification in patients with SCAI Stage C CS for clinical outcomes including LVAD, OHT, or death at 1 year.

## Graphical abstract

**Graphical Abstract:**
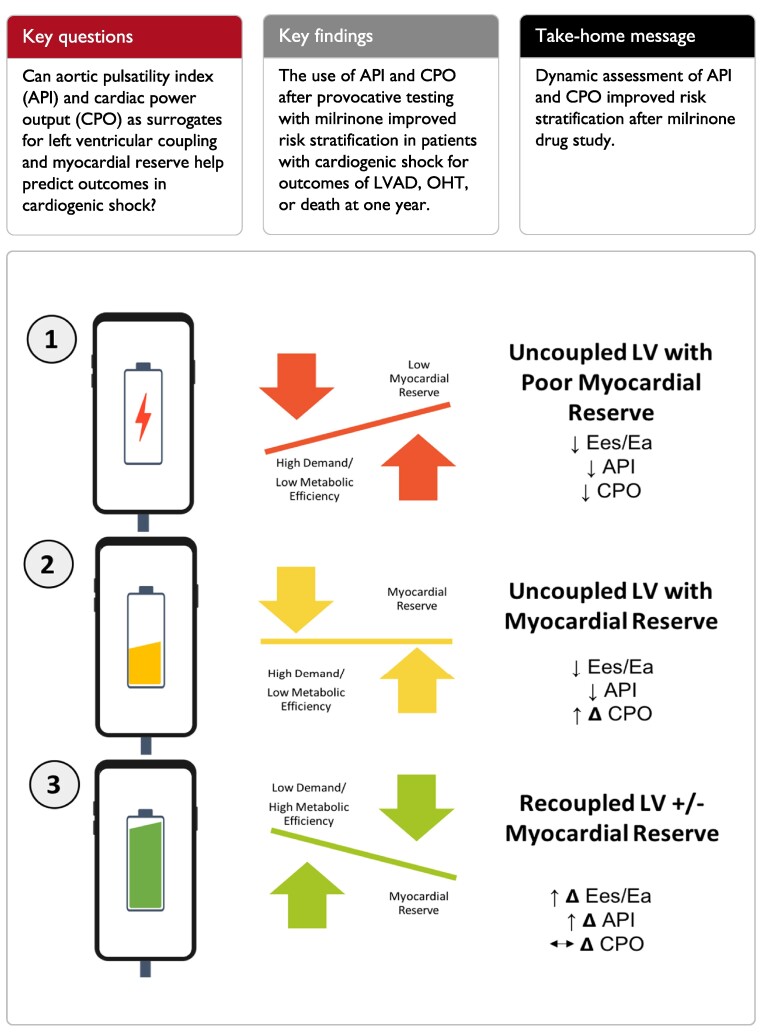

## Introduction

The aortic pulsatility index (API), calculated as (systolic − diastolic blood pressure)/pulmonary capillary wedge pressure (PCWP), is an advanced haemodynamic variable representing both cardiac filling pressures and contractility. Aortic pulsatility index is significantly associated with clinical outcomes in patients with decompensated heart failure and cardiogenic shock (CS).^1,2^ Aortic pulsatility index is a left-sided analogue to the pulmonary artery pulsatility index (PAPI), which, when low, is also prognostic for major adverse cardiovascular events.^3^ Cardiac power output (CPO), another advanced haemodynamic variable, is calculated as cardiac output (CO) multiplied by mean arterial pressure (MAP), and divided by 451. It is prognostic for patients presenting with CS in the setting of acute myocardial infarction, although its prognostic potential has recently been challenged in CS secondary to heart failure.^2,4–7^

Left ventricular (LV) coupling, also known as ventriculo-arterial coupling, refers to the heart’s ability to pump in relation to the load opposed by the arterial system.^8^ The slope of the end-systolic ventricular pressure–volume relationship, known as the end-systolic elastance (Ees), is the maximal ventricular elastance and represents LV contractility. The effective arterial elastance (Ea) represents arterial afterload and all extracardiac forces opposing LV contractility. Left ventricular coupling can therefore be analysed as the ratio of Ees to Ea gathered from specialized catheters that can simultaneously measure intracavitary pressure and volume to generate pressure–volume loops. However, a method to calculate LV coupling with pulmonary artery (PA) catheter and haemodynamic monitoring has not been established, limiting the utilization of the coupling ratio in routine clinical care. Aortic pulsatility index, which incorporates both contractility and afterload strongly correlates with mechanical efficiency, especially at higher filling pressures, can serve as a surrogate for the coupling ratio.^1,5^

Cardiac power output is the product of simultaneously measured cardiovascular flow (i.e. CO) and MAP. In a small study of patients in CS, CPO was used as a surrogate for myocardial reserve—patients who demonstrated a response to incremental dobutamine infusions with an increase in CPO had better survival.^9^ The ability to augment CPO represents mechanical or contractile reserve. When contractile reserve is assessed non-invasively, patients with adequate reserve have improved outcomes across the entire clinical spectrum of heart failure.^10,11^ Contractile reserve implies sufficient metabolic reserve. Patients with metabolic reserve have additional energy sources at their disposal and generate sufficient adenosine triphosphate to allow for progression of the cardiac cycle and generation of contraction forces.^12,13^ Thus, changes in CPO reflect overall myocardial reserve, which includes both metabolic and mechanical reserve.

### Hypothesis and purpose

We investigated the utility of API and CPO as surrogates for LV coupling and myocardial reserve, after provocative testing with milrinone, as a way to risk-stratify patients in CS and their clinical outcomes.

## Methods

This study was approved by our hospital’s Institutional Review Board. Data were retrospectively collected on consecutive patients undergoing right heart catheterization (RHC) at a tertiary academic medical centre between January 2013 and November 2019. Patients underwent a milrinone drug study to assess inotropic response in patients with concern for SCAI Stage C CS [PCWP of ≥15 mmHg and/or a mean pulmonary arterial pressure of >25 mmHg with a cardiac index (CI) of <2.2 L/min/m^2^] as previously described.^1^ The milrinone drug study consisted of a 50 mcg/kg milrinone bolus infused over 10 min. Haemodynamic measurements were obtained at baseline and after the milrinone bolus. No other interventions were administered prior to the repeat haemodynamic measurements post-milrinone drug study. Continuation of milrinone after the drug study was at the discretion of the treating physician.

Patients included were ≥18 years of age, not on vasoactive therapy at baseline, and not receiving temporary or durable mechanical circulatory support. Patients were excluded if there was concern for acute myocardial infarction CS. Baseline demographics, medical diagnoses, and heart failure-specific guideline-directed medical therapies were collected. Right heart catheterization measurements included all standard haemodynamics. Advanced haemodynamic variables were then calculated, including API, CPO, LV stroke work index (LVSWI), RV stroke work index (RVSWI), and PAPI. In addition, we derived variations of CPO, including right atrial (RA) pressure (e.g. CPO-RA), cardiac power index (CPI), and CPI-RA.

An API < 1.45, previously identified as the optimal threshold from receiver-operating characteristic (ROC) curve analysis in the same cohort, was utilized to define baseline LV uncoupling.^1^ Re-coupling was then identified after ROC analysis by the Youden index method which revealed an optimal final API cut-off following milrinone bolus for the composite primary outcome [e.g. incidence of LV assist device (LVAD), orthotopic heart transplantation (OHT), or death at 1 year]. Patients who recoupled were then further stratified by those with improvements myocardial reserve, or not, as determined by significant changes in CPO similarly determined by optimal final CPO cut-off by ROC analysis for the composite primary outcome.

Individual patient outcomes were recorded and stratified by medical management with guideline-directed medical therapy, inotropic therapy, need for advanced heart failure therapies including LVAD implantation or OHT, or death at 30 days and 1 year.

### Statistics

Continuous variables were summarized as means and standard deviations if normally distributed by the Shapiro–Wilk test or alternatively as medians and interquartile ranges. Comparison of continuous variables was performed with either Student’s *t*-tests or Mann–Whitney *U* tests, respectively. Categorical variables were reported as relative counts and corresponding frequencies, which were compared using *χ^2^* tests of association. Two-sided *P-*values below 0.05 were considered statistically significant. Receiver-operating characteristic curve analysis was employed to identify the optimal cut-off by the Youden index method for API and CPO after milrinone infusion (representing re-coupling and myocardial reserve, respectively) using the primary endpoint of probability of LVAD, OHT, or death at 1 year. The Kaplan–Meier curves were generated with time to LVAD, OHT, or death. All statistical analyses were performed using Stata version 17.0 (StataCorp, College Station, TX, USA).

## Results

### Baseline characteristics

A total of 224 patients with concern for acute or acute on chronic SCAI Stage C CS underwent RHC with milrinone drug study. At the time of the RHC and drug study, there was no concern for acute myocardial infarction CS. At the time of the procedure, the average age was 57 years (48–66 years), 34% were women, 39% white, and 31% had ischaemic cardiomyopathy. Of 224 total patients, 117 patients had a baseline API < 1.45. Receiver-operating characteristic analysis revealed an optimal API cut-off after milrinone infusion of 2.2 based on the Youden Index. Similarly, the optimal cut-off for baseline CPO was 0.64 W and after milrinone infusion was 0.77 W. Of the 117 uncoupled patients, 88 patients (75%) had a final API < 2.2 after milrinone load and 29 patients (25%) had a final API ≥ 2.2 after milrinone load. The baseline characteristics and pre-drug study medications, separated by final API post-milrinone drug study, are listed in *Table 1*. Patients with recoupled LVs (final API ≥ 2.2) were more likely to be male (93 vs. 69%, *P* = 0.010) and less likely to be on beta-blockers (69 vs. 86%, *P* = 0.030).

**Table 1:** Baseline characteristics for patients in cardiogenic shock with low aortic pulsatility index (aortic pulsatility index < 1.45) separated by their ability to demonstrate left ventricular recoupling

	Final API < 2.2 (*n* = 88)	Final API ≥ 2.2 (*n* = 29)	*P*-value
Demographics			
Age, years	52.4 ± 12.6	53.0 ± 16.1	0.860
Sex, male	**61 (69.3%)**	**27 (93%)**	**0**.**010**
Race/ethnicity			0.320
Caucasian	35 (39.7%)	7 (24.1%)	
Black	44 (50.0%)	18 (62.1%)	
Asian	2 (2.3%)	0 (0%)	
Other	7 (8.0%)	4 (13.8%)	
Comorbidities			
Hypertension	46 (52.2%)	15 (52.0%)	0.960
Ischaemic cardiomyopathy	28 (31.8%)	6 (20.7%)	0.250
Coronary artery disease	29 (33.0%)	10 (34.5%)	0.880
Hyperlipidaemia	29 (33.0%)	8 (27.6%)	0.590
Atrial fibrillation	27 (30.7%)	13 (44.8%)	0.160
Diabetes mellitus	36 (41.0%)	9 (31.0%)	0.340
COPD	7 (8.0%)	2 (6.9%)	0.850
Peripheral arterial disease	2 (2.3%)	0 (0%)	0.410
Stroke/TIA	9 (10.2%)	1 (3.4%)	0.260
Chronic kidney disease	34 (38.6%)	15 (51.7%)	0.220
Left ventricular ejection fraction	20.6 ± 9.0	22.1 ± 9.0	0.440
Pre-study medications			
Beta-blocker	**76 (86.3%)**	**20 (69.0%)**	**0**.**030**
ACEi, ARB, and ARNi	58 (65.9%)	18 (62.1%)	0.710
MRA	50 (56.8%)	21 (72.4%)	0.140
Hydralazine	5 (5.7%)	4 (13.8%)	0.160
Isordil	4 (4.5%)	2 (6.9%)	0.620
Digoxin	17 (19.3%)	10 (34.5%)	0.090

Bold font denotes significant values.

### Pre- and post-milrinone haemodynamics

For baseline haemodynamics assessed before milrinone drug study (*Table 2*), patients with LV recoupling (final API ≥ 2.2) post-milrinone had lower RA pressure (11.4 ± 5.1 vs. 17.5 ± 6.8, *P* < 0.001), higher CI by thermodilution (1.86 ± 0.61 vs. 1.54 ± 0.46, *P* = 0.010), higher PA saturation (53.5 ± 7.2 vs. 47.7 ± 9.9, *P* = 0.004), higher API (1.1 ± 0.2 vs. 0.96 ± 0.27, *P* = 0.02), higher CPO (0.7 ± 0.22 vs. 0.62 ± 0.17, *P* = 0.05), higher PAPI (3.0 ± 1.8 vs. 1.8 ± 0.9, *P* < 0.001), and lower RA:PCWP ratio (0.40 ± 0.15 vs. 0.58 ± 0.2, *P* < 0.0001). For post-milrinone haemodynamics, patients with LV recoupling (final API ≥2.2) had lower RA pressure (7.4 ± 4.0 vs. 13.4 ± 5.8, *P* < 0.0001), lower systolic PA pressure (44.0 ± 13.9 vs. 57.4 ± 13.5, *P* < 0.0001), lower diastolic PA pressure (21.9 ± 7.0 vs. 30.5 ± 8.1, *P* < 0.0001), lower mean PA pressure (29.6 ± 9.0 vs. 40.2 ± 9.2, *P* < 0.0001), lower PCWP (14.1 ± 5.6 vs. 26.2 ± 7.5, *P* < 0.0001), higher PA saturation (67.1 ± 5.8 vs. 61.8 ± 9.3, *P* = 0.006), higher pulse pressure (44.1 ± 13.3 vs. 35.5 ± 9.0, *P* = 0.0001), higher API (3.5 ± 1.4 vs. 1.4 ± 0.4, *P* < 0.0001), higher CPO (1.03 ± 0.39 vs. 0.85 ± 0.28, *P* = 0.008), higher LVSWI (29.8 ± 11.4 vs. 21.2 ± 8.2, *P* < 0.0001), higher PAPI (3.7 ± 2.7 vs. 2.5 ± 2.2, *P* = 0.03), lower PA elastance (0.7 ± 0.27 vs. 1.1 ± 0.6, *P* = 0.0002), and higher PA compliance (3.5 ± 2.2 vs. 2.6 ± 2.1, *P* = 0.05).

**Table 2:** Pre- and post-milrinone haemodynamics for patients in cardiogenic shock with low aortic pulsatility index (aortic pulsatility index < 1.45) separated by their ability to demonstrate left ventricular recoupling

	Baseline haemodynamics			Post-milrinone haemodynamics		
	Final API < 2.2 (*n* = 88)	Final API ≥ 2.2 (*n* = 29)	*P*-value	Final API < 2.2 (*n* = 88)	Final API ≥ 2.2 (*n* = 29)	*P*-value
Right atrial pressure (mmHg)	**17.5 ± 6.8**	**11.4** ± **5.1**	***<0***.***001***	**13.4** ± **5.8**	**7.4** ± **4.0**	***<0***.***0001***
Systolic pulmonary artery pressure (mmHg)	61.1 ± 10.8	59.2 ± 12.4	*0*.*45*	**57.4** ± **13.5**	**44.0** ± **13.9**	***<0***.***0001***
Diastolic pulmonary artery pressure (mmHg)	34.1 ± 7.2	31.4 ± 6.3	*0*.*08*	**30.5** ± **8.1**	**21.9** ± **7.0**	***<0***.***0001***
Mean pulmonary artery pressure (mmHg)	44.4 ± 7.7	41.5 ± 8.5	*0*.*09*	**40.2** ± **9.2**	**29.6** ± **9.0**	***<0***.***0001***
Pulmonary capillary wedge pressure (mmHg)	30.5 ± 6.7	28.8 ± 6.7	*0*.*240*	**26.2** ± **7.5**	**14.1** ± **5.6**	***<0***.***0001***
Pulmonary artery saturation (%)	**47.7** ± **9.9**	**53.5** ± **7.2**	***0***.***004***	**61.8** ± **9.3**	**67.1** ± **5.8**	***0***.***006***
Cardiac index, Fick (L/min/m^2^)	1.66 ± 0.4	1.76 ± 0.1	*0*.*170*	2.4 ± 0.6	2.6 ± 0.5	*0*.*07*
Cardiac index, thermodilution (L/min/m^2^)	**1.54** ± **0.5**	**1.86** ± **0.6**	***0***.***010***	N/A	N/A	N/A
Mean arterial pressure (mmHg)	84.0 ± 11.7	85.4 ± 13.6	*0*.*580*	80.1 ± 14.6	81.4 ± 15.4	*0*.*68*
Pulse pressure (mmHg)	28.8 ± 8.9	31.3 ± 8.3	*0*.*180*	**35.5** ± **9.0**	**44.1** ± **13.3**	***0***.***0001***
Heart rate (b.p.m.)	81.8 ± 16.0	86.5 ± 16.4	*0*.*180*	85.4 ± 16.2	84.6 ± 8.3	*0*.*81*
Systemic vascular resistance (dyn/s/cm^−5^)	1670 ± 554	1647 ± 589	*0*.*850*	1240 ± 463	1037 ± 343	*0*.*08*
Pulmonary vascular resistance (Wood units)	4.4 ± 2.2	3.7 ± 2.1	*0*.*14*	3.2 ± 2.0	2.8 ± 1.1	*0*.*32*
Aortic pulsatility index	**0.96** ± **0.27**	**1.1** ± **0.2**	***0***.***02***	**1.4** ± **0.4**	**3.5** ± **1.4**	***<0***.***0001***
Cardiac power output (W)	**0.62** ± **0.17**	**0.70** ± **0.22**	***0***.***05***	**0.85** ± **0.28**	**1.03** ± **0.39**	***0***.***008***
Left ventricular stroke work index (g/m^2^/beat)	15.2 ± 5.2	15.3 ± 6.6	*0*.*960*	**21.2** ± **8.2**	**29.8** ± **11.4**	***<0***.***0001***
Right ventricular stroke work index (g/m^2^/beat)	7.6 ± 3.1	8.6 ± 3.0	*0*.*140*	10.5 ± 5.1	9.7 ± 4.4	*0*.*47*
Pulmonary artery pulsatility index	**1.8** ± **0.9**	**3.0** ± **1.8**	***<0***.***001***	**2.5** ± **2.2**	**3.7** ± **2.7**	***0***.***03***
Right atrial: pulmonary capillary wedge pressure ratio	**0.58** ± **0.20**	**0.40** ± **0.15**	***<0***.***0001***	0.52 ± 0.19	0.55 ± 0.29	*0*.*62*
Pulmonary artery elastance (mmHg/mL)	1.6 ± 0.7	1.4 ± 0.5	*0*.*26*	**1.1** ± **0.6**	**0.7** ± **0.27**	***0***.***0002***
Pulmonary arterial compliance (mL/mmHg)	1.7 ± 1.0	1.8 ± 1.0	*0*.*94*	**2.6** ± **2.1**	**3.5** ± **2.2**	***0***.***05***

Bold font denotes significant values.

### Static assessment of aortic pulsatility index and cardiac power output

**Figure 1:**
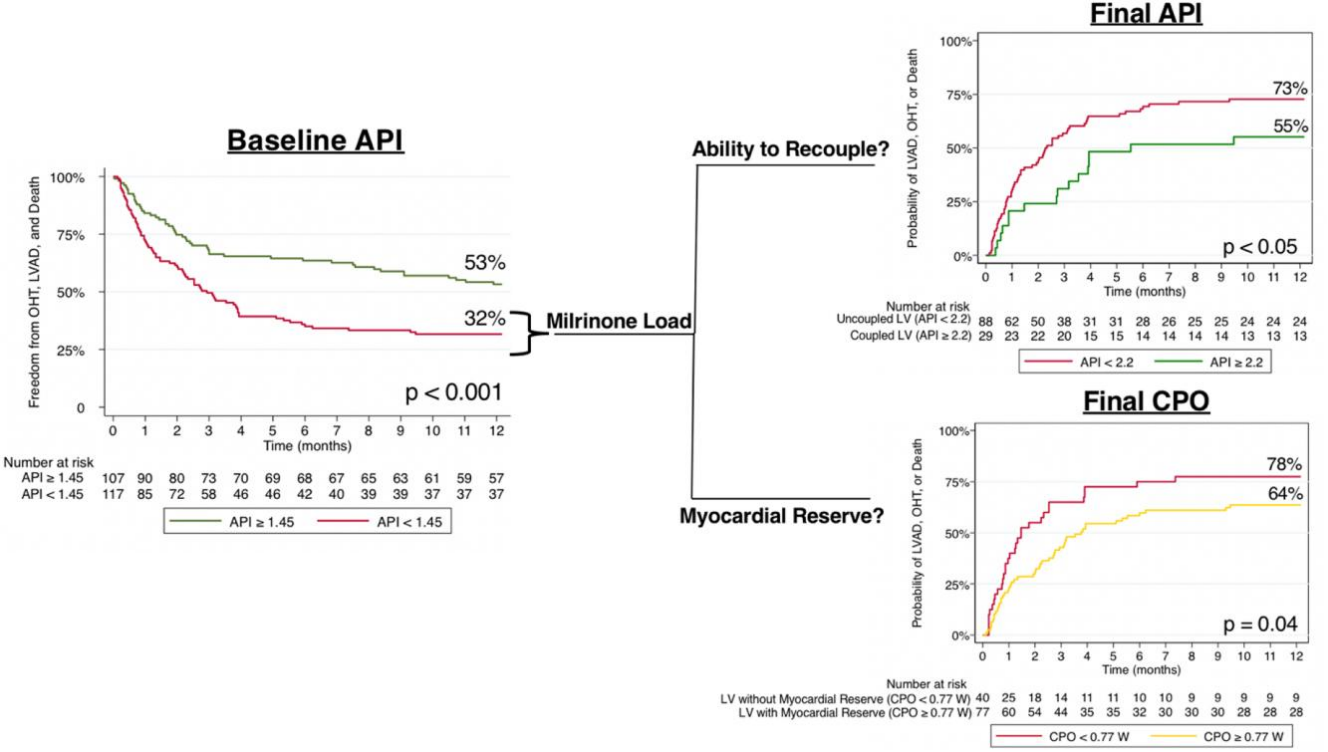
Dynamic assessment of left ventricular coupling with aortic pulsatility index and myocardial reserve with cardiac power output improves risk stratification compared with static baseline haemodynamics and predicts outcomes of left ventricular assist device, orthotopic heart transplantation, or death at 1 year in patients with cardiogenic shock. Static assessment with baseline aortic pulsatility index ≥ 1.45 and baseline aortic pulsatility index < 1.45 (figure on the left). Dynamic assessment after milrinone with uncoupled left ventricle (final aortic pulsatility index < 2.2) and coupled left ventricle (final aortic pulsatility index ≥ 2.2). Dynamic assessment of left ventricle after milrinone without myocardial reserve (final cardiac power output W < 0.77) and with myocardial reserve (final cardiac power output≥ 0.77 W). API, aortic pulsatility index; CPO, cardiac power output; LVAD, left ventricular assist device; OHT, orthotopic heart transplantation.

**Figure 2:**
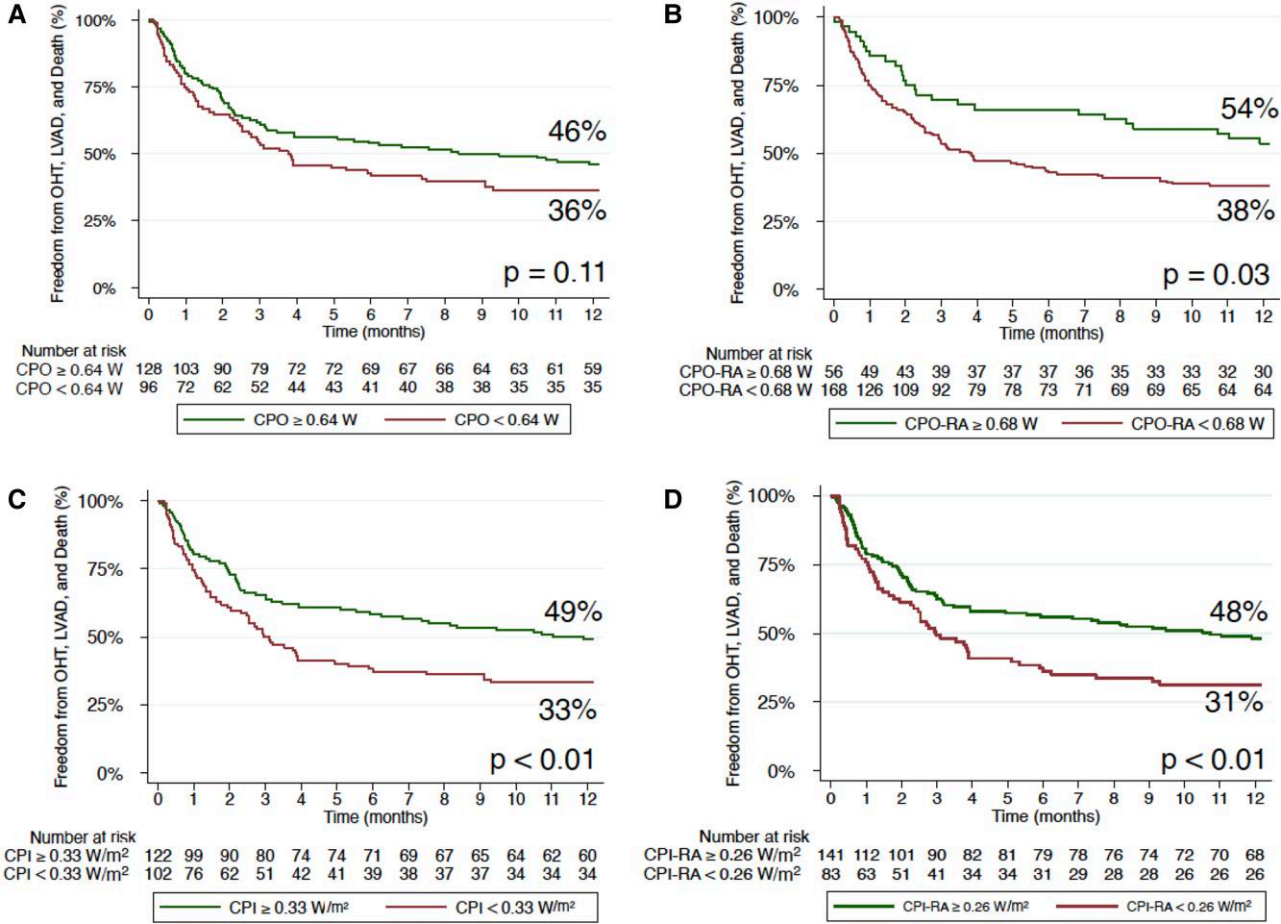
Static assessment of cardiac power output, cardiac power output-right atrial pressure, cardiac power index, and cardiac power index-right atrial pressure at baseline in patients with cardiogenic shock before milrinone bolus with composite endpoint of freedom from orthotopic heart transplantation, left ventricular assist device, or death at 1 year. (*A*) Baseline low cardiac power output < 0.64 W and baseline high cardiac power output ≥ 0.64 W; non-significant, *P* = 0.11. (*B*) Baseline low cardiac power output-right atrial pressure < 0.68 W and baseline high cardiac power output-right atrial pressure ≥ 0.68 W; significant, *P* = 0.03. (*C*) Baseline low cardiac power index < 0.33 W/m^2^ and baseline high cardiac power index ≥ 0.33 W/m^2^; significant, *P* < 0.01. (*D*) Baseline low cardiac power index-right atrial pressure < 0.26 W/m^2^ and baseline high cardiac power index-right atrial pressure ≥ 0.26 W/m^2^; significant, *P* < 0.01. CPO, cardiac power output; RA, right atrial pressure; CPI, cardiac power index; LVAD, left ventricular assist device; OHT, orthotopic heart transplantation.

**Figure 3:**
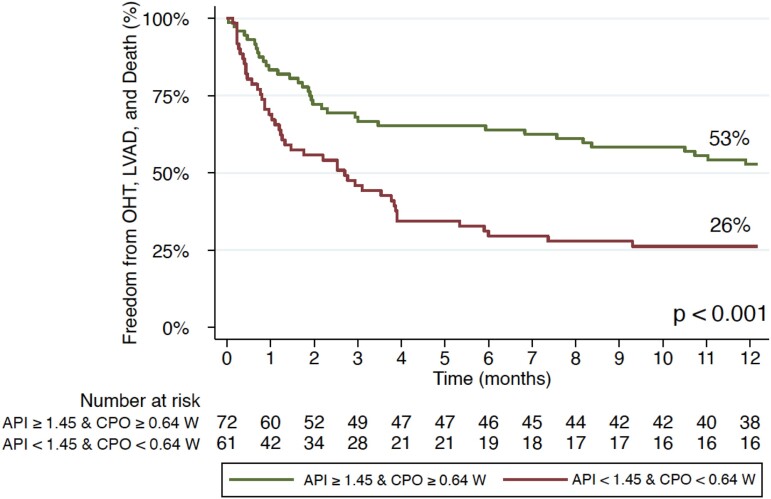
Static assessment of combined baseline aortic pulsatility index and cardiac power output in patients with cardiogenic shock before milrinone bolus with composite endpoint of freedom from OHT, LVAD, or death at 1 year. Low baseline aortic pulsatility index and cardiac power output (<1.45 and <0.64 W, respectively) and high baseline aortic pulsatility index and cardiac power output (≥ 1.45 and ≥0.64 W, respectively); *P* < 0.001. API, aortic pulsatility index; CPO, cardiac power output; LVAD, left ventricular assist device; OHT, orthotopic heart transplantation.

Of the 224 patients who underwent static assessment prior to milrinone bolus, 117 patients had low baseline API <1.45 and 107 patients had high baseline API ≥ 1.45. Of the high baseline API patients, 53% of patients met the composite endpoint of freedom from LVAD, OHT, or death at 1 year vs. only 32% of patients with low baseline API (*P* < 0.001; *Figure 1*). Of these patients, 128 patients had high baseline CPO ≥0.64 W and 96 patients had low baseline CPO <0.64 W. Of the patients with high baseline CPO, 48% met the composite endpoint of freedom from LVAD, OHT, or death at 1 year vs. 36% of patients with low baseline CPO, but this was not significant (*P* = 0.11; *Figure 2A*). However, when RA pressure is taken into account, 54% of patients with high CPO-RA had freedom from the composite endpoint, whereas only 38% of patients did with low CPO-RA which was significant (*P* = 0.03; *Figure 2B*). Likewise, with CPI and CPI-RA, there was a significant difference in freedom from the composite endpoint (high CPI—49% vs. low CPI—33%; *P* < 0.01) and (high CPI-RA—48% vs. low CPI-RA—31%; *P* < 0.01; *Figure 2C* and *D*). Static assessment of combined high baseline API and CPO vs. low baseline API and CPO was significant with 55 vs. 26% meeting the composite endpoint of freedom from LVAD, OHT, or death at 1 year, respectively (*P* < 0.001; *Figure 3*).

### Dynamic assessment of aortic pulsatility index as a surrogate for left ventricular coupling

Of the 117 patients with baseline API <1.45, 88 patients (75%) had a final API <2.2 after milrinone load, consistent with persistent decoupling of the LV. Of these patients, 73% met the composite endpoint of LVAD, OHT, or death at 1 year. The remaining 29 patients (25%) had a final API ≥ 2.2 post-milrinone drug study consistent with the ability to recouple their LVs. Of these patients, only 55% met the composite endpoint of LVAD, OHT, or death at 1 year (*P* = 0.046; *Figure 1*). One-year mortality for final API ≥ 2.2 was 2 deaths (6.9%) vs. final API <2.2 was 17 deaths (19.3%), *P* = 0.120. Thirty-day composite endpoint for final API < 2.2 (40.9 ± 5.2%) vs. final API ≥ 2.2 (24.1 ± 8.1%), *P* = 0.100. Thirty-day mortality for final API ≥ 2.2 was 1 death (3.4%) vs. final API <2.2 was 6 deaths (6.8%), *P* = 0.500.

### Dynamic assessment of cardiac power output as a surrogate for myocardial reserve

Of the 117 patients with baseline API < 1.45, CPO was also calculated after milrinone load to distinguish which patients demonstrated myocardial reserve. Forty patients (34%) had low myocardial reserve (final CPO < 0.77 W). Of these patients, 78% met the composite endpoint of LVAD, OHT, or death at 1 year. Of the 77 patients (66%) who demonstrated myocardial reserve (final CPO ≥ 0.77 W), only 64% met the composite endpoint of LVAD, OHT, or death at 1 year (*P* = 0.039; *Figure 1*). The 30-day composite endpoint for final CPO < 0.77 W (52.5 ± 8%) vs. final CPO ≥0.77 W (28.5 ± 5.2%), *P* = 0.010. Thirty-day mortality for final CPO < 0.77 W was 4 deaths (10%) vs. final CPO ≥ 0.77 W was 3 deaths (3.9%), *P* = 0.190. One-year mortality for final CPO < 0.77 W was 9 deaths (22.5%) vs. final CPO ≥0.77 W was 10 deaths (12.9%), *P* = 0.190. For dynamic CPO-RA, 81% of patients with low final CPO-RA met the composite endpoint of LVAD, OHT, or death, whereas only 62% of patients did with high final CPO-RA which was significant (*P* = 0.02; Supplementary material online, *Figure S1*). However, with dynamic final CPI-RA, there was no difference between the two groups (low final CPI-RA, 78% vs. high final CPI-RA, 66%; *P* = 0.11).

### Dynamic assessment of aortic pulsatility index and cardiac power output

**Figure 4:**
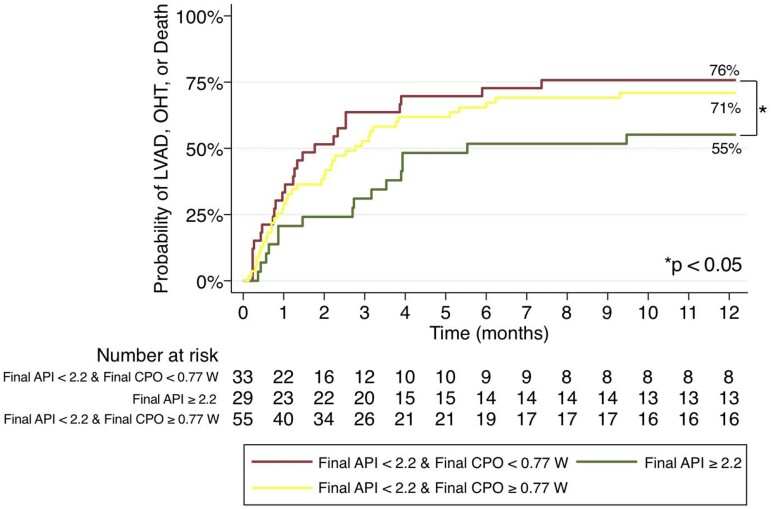
Final aortic pulsatility index and cardiac power output used in combination to further risk-stratify patients in cardiogenic shock with composite endpoint of left ventricular assist device, orthotopic heart transplantation, or death at 1 year. Uncoupled left ventricle without myocardial reserve (final aortic pulsatility index < 2.2 and final cardiac power output <0.77 W); uncoupled left ventricle with myocardial reserve (final aortic pulsatility index < 2.2 and final cardiac power output ≥ 0.77 W); and recoupled left ventricle (final aortic pulsatility index ≥ 2.2). API, aortic pulsatility index; CPO, cardiac power output; LV, left ventricle; LVAD, left ventricular assist device; OHT, orthotopic heart transplantation.

When API and CPO were used in combination to risk-stratify patients, a coupled LV demonstrated the lowest probability of the composite endpoint (55%), whereas CPO only modestly discriminated between uncoupled LVs. Final API < 2.2 and final CPO < 0.77 W had a 76% probability of the composite endpoint, whereas final API < 2.2 and final CPO ≥ 0.77 W had a 71% probability the composite endpoint of LVAD, OHT, or death at 1 year (*P* = 0.49; *Figure 4*). Conversely, patients with a final API ≥2.2 had a 53% composite event rate at 1 year regardless of final CPO (*P* < 0.05).

## Discussion

Advanced haemodynamic variables such as API and CPO are helpful tools in prognosticating patients in CS.^1,4^ Until now, API and CPO have been used as static assessments to predict outcomes. Here, we introduced a framework to understand the dynamic metrics in relation to the physiologic principles of ventricular coupling and myocardial reserve. This is the first study to evaluate these metrics, in tandem, following provocative, haemodynamic manoeuvres in the catheterization lab. With the use of API and CPO in a dynamic assessment, we were able to better risk-stratify patients with SCAI Stage C CS between significant clinical outcomes—progression to LVAD, OHT, or death.

Patients who demonstrated a recoupled LV by a high final API ≥2.2 were more likely to be male and less likely to be on beta-blockers. While beta-blockers can be used while patients are on inotropic therapy with milrinone, in the setting of CS, they are often held due to their negative inotropic effects. This may explain why more patients off of beta-blockers were able to recouple their LVs. Also, patients with recoupled LVs had better baseline right- and left-sided haemodynamics, as demonstrated by lower RA pressure, lower RA:PCWP ratio, and higher PAPI, in addition to higher PA saturation, CI by thermodilution, API, and CPO, respectively. Importantly, multiple surrogates for right-sided dysfunction (e.g. RA, RA:PCWP, and PAPI) were significant in the static assessment and when RA pressure was accounted for with CPO, there was improved risk stratification between groups (e.g. CPO-RA and CPI-RA) similar to previously reported literature.^14,15^ After the milrinone drug study, patients with recoupled LVs continued to demonstrate improvement in both their right- (e.g. lower RA pressure, lower PA pressures, higher PAPI, lower PA elastance, and higher PA compliance) and left-sided haemodynamics (e.g. lower PCWP, higher PA saturation, higher pulse pressure, higher API, higher CPO, and higher LVSWI). Notably, PAPI, PA elastance, and PA compliance were significantly improved after milrinone bolus in the patients who recoupled their LVs which likely represents concomitant RV recoupling—these metrics have also been shown to be predictors of outcomes in CS.^16^

It is known that patients with uncoupled LVs (low baseline API and Ees/Ea) with poor myocardial reserve (low baseline CPO) have the worst outcomes.^1,2,4^ We further validate this literature with a static assessment of combined low API and CPO which had a much lower composite endpoint of freedom from LVAD, OHT, or death at 1 year than combined high API and CPO. We expand upon the previous literature by demonstrating that patients with uncoupled LVs have better outcomes after dynamic assessment with milrinone load if they are able to demonstrate LV recoupling (high final API) and/or myocardial reserve (high final CPO). Patients who demonstrated LV recoupling by API had a lower composite endpoint with the incidence of LVAD, OHT, or death at 1 year. There was no difference in mortality alone at 30 days, but there was a trend towards lower mortality at 1 year with recoupled LVs. Similarly, patients who demonstrated myocardial reserve by CPO also had a lower composite endpoint of LVAD, OHT, or death at 1 year. The significance was even demonstrated as early as 30 days. There was no difference in mortality alone at 30 days or 1 year. When RA pressure was used in the calculation for dynamic CPO (e.g. final CPO-RA), there remained a significant difference in the composite endpoint (e.g. incidence of LVAD, OHT, or death at 1 year); however, this was not seen with final CPI-RA.

**Figure 5:**
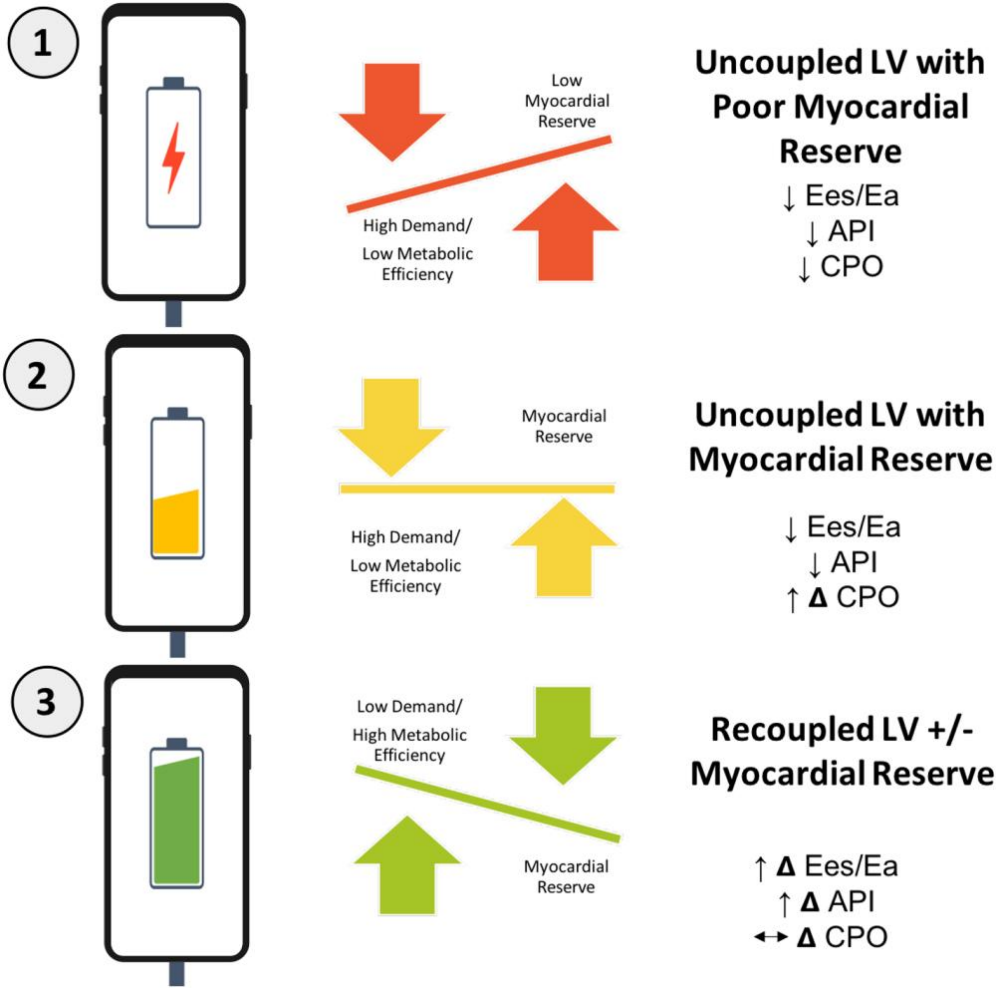
Patients in cardiogenic shock with uncoupled left ventricles (low baseline aortic pulsatility index and Ees/Ea) with poor myocardial reserve (low baseline cardiac power output) have the worst outcomes [denoted by a low battery]. These patients with uncoupled left ventricles have better outcomes after dynamic assessment with milrinone load if they are able to demonstrate myocardial reserve [high final cardiac power output; denoted by a battery that is not fully charged]. Patients in cardiogenic shock have the best outcomes if they demonstrate a recoupled left ventricle (high final aortic pulsatility index) with or without myocardial reserve (final cardiac power output), although there is a trend towards better outcomes if these patients also have myocardial reserve [denoted by a fully charged battery]. API, Aaortic pulsatility index (API); CPO, cardiac power output; Ea, effective arterial elastance; Ees, end-systolic elastance; LV, left ventricle.

When assessing the dynamic effects of API and CPO in combination, patients had the best outcomes if they demonstrated LV recoupling with myocardial reserve. While final CPO was unable to further discriminate between persistently uncoupled LVs, there was a trend towards better outcomes if these patents also had myocardial reserve. The number of patients in this subcohort may be too small to see a difference, but in a larger patient population, this could be clinically meaningful. Dynamic changes in CPO reflect metabolic reserve and higher energy stores and a high final API reflects the ability of the LV to recouple signifying improved mechanical efficiency of energy transfer.^17^ During LV recoupling, an uncoupled LV goes from a high demand state (e.g. elevated preload) with low mechanical efficiency (e.g. narrow pulse pressure) to a coupled LV with low demand (e.g. lower preload) and high mechanical efficiency (e.g. wider pulse pressure). Taken together, they provide valuable information on the energy stores and efficient transfer of mechanical energy in the heart (*Figure 5*).

While outcomes for patients with decompensated heart failure have improved throughout the years with guideline-directed medical therapy, the long-term prognosis still remains poor overall.^18^ For patients in CS in the current era, complete PA catheter haemodynamics are associated with improved survival.^19^ However, invasive haemodynamics represent a moment in time and it remains difficult for these studies to translate data on how these individual patients may progress to requiring advanced therapies. Assessing myocardial response to provocative manoeuvres can provide additional prognostic information that can help guide management. Specifically, dynamic assessment of API and CPO was able to better risk-stratify patients with SCAI Stage C CS by clinical outcomes. Importantly, as noted by the high rates of advanced therapies and mortality in this CS cohort, clinically these patients would benefit from early referral for evaluation of advanced therapies.

### Limitations

This is a retrospective study at a single institution. The sample size for our sub-analysis was smaller, given that we investigated a sicker cohort of patients in CS with low baseline API.

## Clinical perspective

Our research study highlights improved risk stratification of patients in SCAI Stage C cardiogenic shock (CS) when utilizing a dynamic assessment of haemodynamics with provocative manoeuvres as opposed to only static measurements. Using aortic pulsatility index as a surrogate for left ventricular (LV) coupling and cardiac power output as a surrogate for myocardial reserve after milrinone bolus improved risk stratification for patients in CS.

The clinical implications are to aid physicians in deciding which patients may need more upfront aggressive therapeutics, such as temporary mechanical circulatory support, more urgent evaluation for advanced therapies, or palliative discussions. Patients in CS who can demonstrate LV coupling with myocardial reserve after milrinone bolus may have more time to be optimized medically whereas those with uncoupled LVs and poor myocardial reserve need urgent escalation of care.

## Conclusions

The use of API and CPO in a dynamic assessment after provocative manoeuvres in the catheterization lab led to improved risk stratification in patients with SCAI Stage C CS for clinical outcomes including LVAD, OHT, or death at 1 year.

## Lead author biography


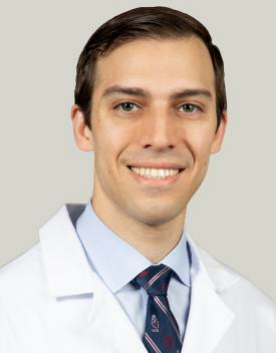


Dr Anthony J. Kanelidis is an Assistant Professor of Medicine in Advanced Heart Failure, Mechanical Circulatory Support, and Transplant Cardiology at the University of Chicago, pursuing research in advanced haemodynamic variables in patients with heart failure and cardiogenic shock. He received his undergraduate degree and medical degree from the University of Miami. He then completed his Internal Medicine residency, Cardiology fellowship, and Advanced Heart Failure and Transplant Cardiology fellowship at the University of Chicago. During this time, he also completed a Clinical Medical Ethics fellowship through the MacLean Center.

## Supplementary Material

oeae072_Supplementary_Data

## Data Availability

The data underlying this article will be shared on reasonable request to the corresponding author.
