# Supplementary material for: Dynamic assessment of left ventricular coupling and myocardial reserve in patients with cardiogenic shock
Source: Eur Heart J Open. 2024 Aug 26;4(5):oeae072. doi: 10.1093/ehjopen/oeae072 (PMC11425697; doi:10.1093/ehjopen/oeae072)
Supplement: oeae072_Supplementary_Data [file oeae072_supplementary_data.pdf]

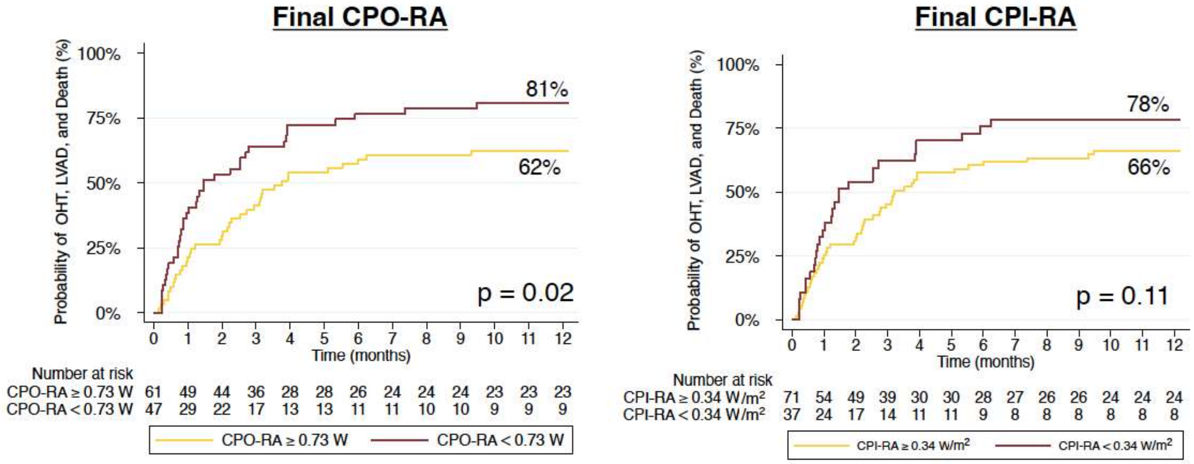

Supplemental Figure. Dynamic assessment of CPO-RA and CPI-RA in patients with cardiogenic shock after milrinone bolus with composite endpoint of risk of OHT, LVAD or death at one year. 81% of patients with low final CPO-RA (red line) met the composite endpoint vs 62% of patients with high final CPO-RA (yellow line) which was significant (P = 0.02). 78% of patients with low final CPI-RA (red line) met the composite endpoint vs 66% of patients with high final CPI-RA (yellow line) which was not significant (P = 0.11). Abbreviations: Cardiac power output (CPO); Right atrial pressure (RA); Cardiac power index (CPI); Left ventricular assist device (LVAD); Orthotopic heart transplantation (OHT).
